# Virtual Reality–Based Training in Radiologic Technology for Contrast-Enhanced Computed Tomography Brain Imaging: Randomized Controlled Trial

**DOI:** 10.2196/88735

**Published:** 2026-05-26

**Authors:** Jongwat Cheewakul, Natee Ina, Pichapat Kanpanluek, Suphalak Khamruang Marshall, Sitthichok Chaichulee

**Affiliations:** 1 Department of Radiology, Faculty of Medicine, Prince of Songkla University, Hat Yai, Songkhla, Thailand; 2 Department of Biomedical Sciences and Biomedical Engineering, Faculty of Medicine, Prince of Songkla University, 15 Kanchanavanich Road, Hat Yai, Songkhla, 90110, Thailand, 66 74451743

**Keywords:** radiologic technology education, virtual reality, immersive learning, computed tomography, 360-degree video, instructional design

## Abstract

**Background:**

Radiologic technology (RT) education faces challenges in bridging theory and practice due to limited clinical opportunities. While virtual reality (VR) enables safe and repeatable practice, a systematic instructional design framework is needed to develop scalable, procedure-focused modules.

**Objective:**

This study evaluates the Radiologic Technology Virtual Reality (RTVR) framework that integrates 360-degree video capture, instructional overlays, interactive assets, and an immersive content authoring platform to deliver a contrast-enhanced computed tomography (CECT) brain scan module.

**Methods:**

In this open-label, parallel-group, randomized controlled trial, 36 year-2 and year-3 RT students with no prior clinical training in diagnostic radiology at a university hospital in Thailand were randomly allocated (1:1) to a VR group or a conventional document-based instruction (control) group. The VR group completed the VR module, a grounded instructional design framework using 360-degree videos and a structured prebrief and debrief, for 20 minutes using a head-mounted display. The control group studied standard curriculum materials for the same duration. Blinding of participants was not possible. Outcome assessment was blinded. The primary outcome was declarative knowledge gain, assessed using a 20-item multiple-choice test before and after intervention. Secondary outcomes included technology acceptance, student satisfaction, and physiological responses during VR immersion.

**Results:**

All 36 randomized participants (VR: n=18, control: n=18) completed the study and were included in the analysis. Experts validated the module as suitable and highly appropriate. Students reported high technology acceptance and satisfaction. Both VR and conventional methods produced substantial gains in declarative knowledge. No statistically significant difference in knowledge gain was detected between groups (test × group: unstandardized regression coefficient β=.056, 95% CI −1.360 to 1.473, *P*=.94). Year-2 students, who had less prior clinical exposure, showed larger pretest to posttest knowledge gains compared to year-3 students. Physiological monitoring showed a reduction in heart rate across the session, while blood pressure remained stable. No adverse events or VR-related discomfort requiring discontinuation was observed.

**Conclusions:**

The RTVR framework, which uses a real 360-degree video of authentic clinical settings, offers a scalable approach to procedural VR content creation without requiring specialist technical skills, distinguishing it from prior VR studies in radiography. These findings support the RTVR framework as a feasible, evidence-informed supplement to RT curricula for knowledge-focused procedural teaching, with learning outcomes comparable to those of conventional instruction in this context.

## Introduction

### Background

Education in radiologic technology (RT) requires preparing students for simple to complex clinical procedures across a range of modalities [1]. The “see one, do one, teach one” apprenticeship model is increasingly difficult to sustain because of patient safety concerns, the high cost and limited availability of advanced imaging equipment, and the need for standardized procedural training [2]. Students often struggle to bridge the theory-practice gap when moving from textbook knowledge to the fluid, real-time application of that knowledge in a busy imaging department [3]. Conventional methods, such as lectures and reading materials, are effective for teaching declarative knowledge but do little to develop the procedural, spatial, and affective skills (eg, patient communication and team coordination) required for clinical competence. This highlights the need for training environments that are safe, repeatable, and realistic [4].

Virtual reality (VR) and augmented reality are now widely used in medical simulation. These technologies offer opportunities for repeated practice without clinical risk [5]. However, many VR applications in radiology still focus on narrow tasks, such as 3D anatomy visualization, rather than the procedural workflow [6,7]. In addition, fully synthetic 3D environments may fail to provide the environmental cues and sense of being in a real clinical space, although the environments can enhance presence through learner agency. This concept is related to situated cognition, which emphasizes authentic contexts as essential for transferring learning to practice [8,9]. Therefore, a key gap exists in how best to design and develop immersive modules for RT procedures—specifically, how an instructional design framework can integrate a real-world environment (eg, 360-degree videos) with structured instructional content (eg, labeled overlays, pop-up guides, and step-by-step procedural demonstrations). Such a framework should be technically and logistically scalable within typical educational settings. It should be quick to implement; feasible to deploy locally; and not dependent on extensive hardware, specialized personnel, or other resource-intensive requirements.

### Challenges in RT Education

Undergraduate RT programs need to prepare students with both the theoretical knowledge and the practical imaging skills expected by accrediting bodies and employers [10]. However, curricula have sometimes emphasized academic performance more than hands-on competence [11]. In response, many programs have moved toward competency-based education and constructive alignment, in which learning outcomes, teaching activities, and assessments are explicitly linked to required clinical competencies. Simulation and laboratory sessions are increasingly designed such that each activity targets specific clinical skills and is assessed against those goals [10,11].

At the same time, clinical training opportunities are limited, and patient safety concerns restrict how much practice students can gain with real patients [12,13]. Simulation-based learning has therefore become an important part of undergraduate RT education, providing a safe, controlled environment for skills practice. More recently, digital and immersive tools such as VR, augmented reality, and 360-degree videos have been introduced to complement traditional learning [12]. These innovations aim to better integrate theory with practice, improve student engagement, and ultimately produce workforce-ready graduates with strong clinical competencies [10-13].

### Virtual Reality and Immersive Media in RT Training

Recent studies show that VR can enhance RT education by providing immersive, hands-on practice, thus improving student learning and confidence [12-15].

O’Connor et al [16] introduced a 3D VR simulation for first-year radiography students, with virtual X-ray examinations delivered via an HTC Vive system. Most students reported enjoying the training and feeling more confident. However, students noted technical issues and the inability to communicate with the virtual patient as limitations. The study concluded that 3D VR is best used to augment existing clinical skills labs and not to replace them [16]. In a subsequent study, O’Connor and Rainford [17] showed that VR training can translate into objective performance gains. The authors reported that students who received VR practice significantly outperformed a prior non-VR cohort on most clinical skill criteria and showed better understanding of clinical indications and patient communication. The study suggested that well-designed VR can improve real-world radiography performance and may help accelerate RT skill acquisition [17]. However, other studies caution that VR’s benefits may not extend equally to all skill domains, especially tactile skills and patient interaction. Kato et al [18] found that first-year radiography students who trained with VR performed similarly to those using real equipment on some technical tasks but significantly worse on skills requiring palpation and direct patient communication. The authors recommended adding haptics, virtual patient communication, and real-world validation. Overall, VR is a powerful tool for reinforcing positioning and technical competencies [15], but it should be blended with hands-on practice to offset its limitations in simulating patient care.

During the COVID-19 pandemic, when clinic access was restricted, several studies showed that VR and virtual simulations could maintain CT imaging education outcomes. Taylor et al [14] reported that immersive VR can help radiography students meet CT competency requirements, provided it is used in an optimized manner—that is, content is grounded in learning design, feedback is provided, and it is supported by trained faculty and adequate technical resources. In addition to fully immersive interactive VR, 360-degree video experiences have value in RT education. Vu et al [19] developed an interactive virtual tour of a radiology department, letting students explore the environment online before placement. Most students reported increased confidence, and the tour was considered an effective stepping stone when clinical observation is limited.

To sum up, VR and other immersive technologies are increasingly used in RT education, with reported gains in engagement, confidence, and specific clinical skills [16-19]. Students generally respond positively to the interactive, realistic nature of VR, but the literature emphasizes that it is not a panacea and should be targeted to specific learning objectives, combined with other teaching methods, and supported by feedback and debriefing [12-15].

### Instructional Design for VR in RT Education

Integrating VR into RT education requires a clear instructional design framework grounded in constructive alignment and competency-based objectives. VR learning activities and assessments should be explicitly mapped to intended learning outcomes and competencies rather than functioning as isolated technological add-ons [11,14]. Students should be assessed in VR or afterward to verify that targeted skills have been achieved. Consequently, VR practice is best positioned as one component of a broader educational strategy that also includes traditional instruction, supervised clinical experience, mentorship, and formal assessment [11,14].

Instructional design principles such as cognitive load theory also provide guidance for VR scenario development. Sweller [20] suggests that learning environments should minimize extraneous load and optimize germane processing. In VR, this translates into focusing each module on a manageable set of procedural steps, using cues to direct attention to clinically relevant information, and avoiding unnecessary interface complexity. When VR activities are aligned with competency frameworks, embedded in learning cycles, and designed with cognitive load in mind, they are more likely to translate into durable, transferable skills for RT students [15,21].

### VR-Based Learning Frameworks

Across the studies reviewed, VR and immersive media have been positioned in distinct ways. O’Connor et al [16] positioned VR as an add-on to existing clinical skills labs, and O’Connor and Rainford [17] later incorporated it into competency-based assessment. Kato et al [18] treated VR as a possible substitute for equipment-based training but ultimately argued for a blended model that pairs VR with haptics, communication practice, and real-world validation. Taylor et al [14] emphasized the need to anchor VR in learning paradigms, aligned with CT competency requirements, and supported by feedback and faculty development. Vu et al [19] used VR primarily for placement orientation. Taken together, these studies converge on the need for procedure-focused VR modules that are clearly aligned with required competencies, embedded in structured learning and assessment cycles, and implemented as part of an integrated educational strategy.

### Study Objectives

This study introduces the Radiologic Technology Virtual Reality (RTVR) instructional design framework, which integrates 360-degree videos, instructional overlays, interactive 3D assets, and an immersive content authoring platform, by implementing a contrast-enhanced CT (CECT) brain imaging learning module. The primary aim was to determine whether the VR module can support declarative knowledge acquisition about the sequence and rationale of procedural steps. We hypothesized that students using the VR module would demonstrate significant pretest to posttest improvement in knowledge of the CECT brain workflow.

Our contributions are as follows. First, we present the design and implementation of the RTVR framework, a pedagogical stack that creates a procedural simulation. Second, we report a comprehensive evaluation of the framework in the context of a CECT brain scan procedure, including a detailed examination of the null result for between-group differences in knowledge gain as well as an analysis of physiological data collected during the experiments. Third, we discuss the implications of the findings and suggest practical considerations for the use of the RTVR framework in similar procedural training contexts.

## Methods

### RTVR Instructional Design Framework

**Figure 1.**
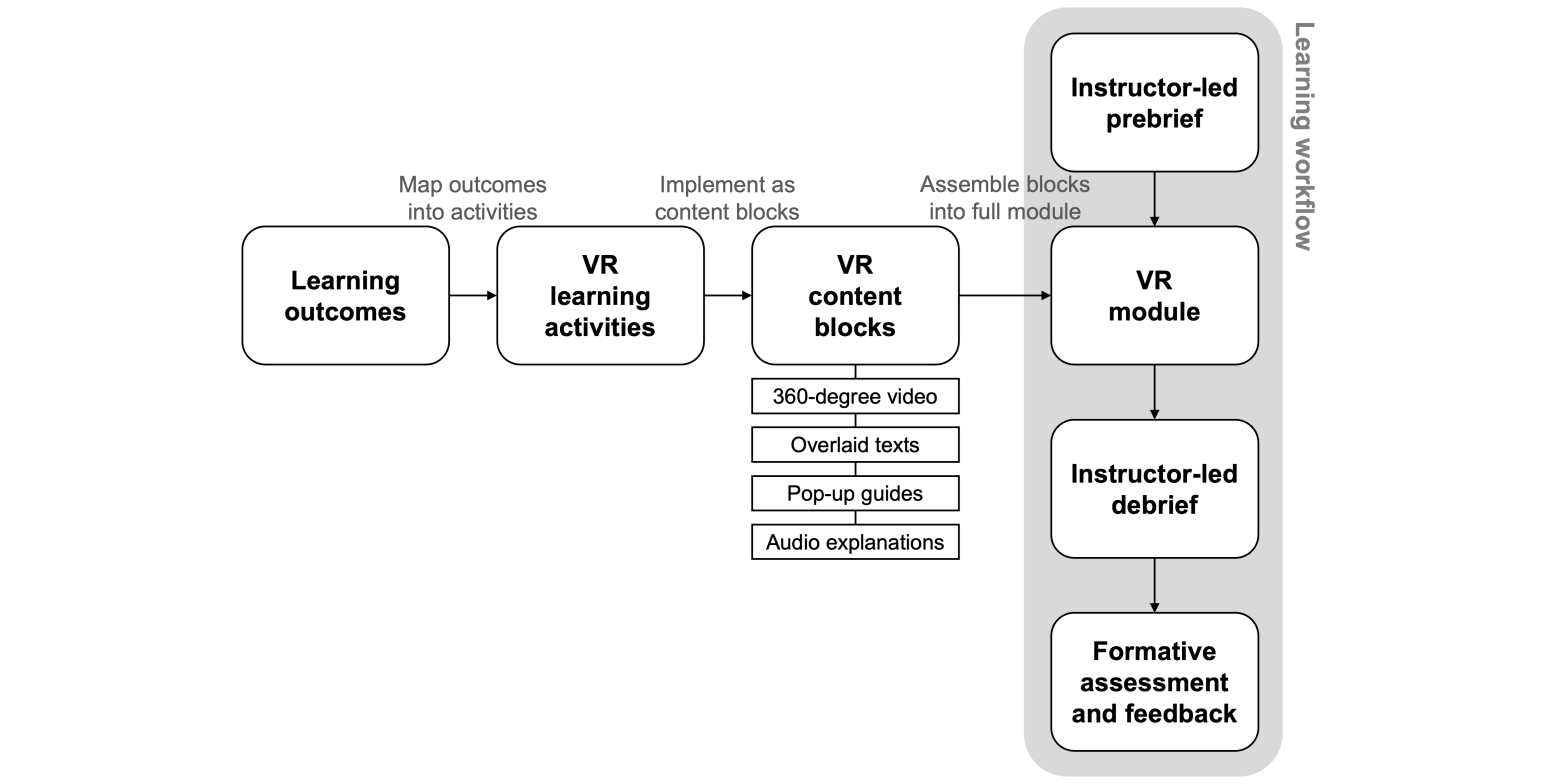
The Radiologic Technology Virtual Reality (RTVR) instructional design framework. Learning outcomes are mapped to learning activities and implemented as content blocks to form the virtual reality (VR) module. The resulting learning workflow (right) frames the VR experience with instructor-led prebriefing, debriefing, and formative assessment.

The RTVR instructional design framework is proposed as a guide for RT educators on how to structure VR learning materials for procedural teaching (see Figure 1). It focuses on how to move from learning outcomes to concrete VR learning activities and then to small content units that can be assembled into a complete module on a platform such as Spatial.io. The goal is that educators can design and develop VR content using familiar pedagogical principles, including constructive alignment and competency-based curriculum design [10,11], without needing specialist skills.

At the *learning outcome layer*, the starting point is the existing RT curriculum [10]. Educators identify the key outcomes for a given procedure, such as screening patients safely, setting up the imaging room, choosing appropriate exposure parameters, or explaining aftercare. Each outcome is then written as a short, observable capability. This step supports constructive alignment. Every subsequent VR element is explicitly tied to a competency that students are expected to demonstrate in clinical placement or real-world scenario [11].

At the *learning activity layer*, each outcome is mapped to 1 or more VR learning activities that reflect how students should encounter and practice the skill. These activities may include (1) concept introduction, which provides an explanation of the key idea or checklist; (2) environment orientation, which shows where equipment and safety features are located; (3) procedural demonstration, which provides a walk-through of the steps and decisions of the procedure; and (4) reflection prompts, which guide students to notice and evaluate specific actions. This structure aligns with established simulation pedagogy that integrates briefing, guided observation, and structured reflection to support effective transfer to practice [4,14].

At the *content block layer*, each activity is implemented as 1 or more small VR content blocks. These content blocks may include (1) 360-degree demonstration videos showing the radiologic technologist’s perspective, (2) still or slow-moving 360-degree views with overlays that label key equipment and areas, (3) pop-up guides and captions that highlight what to look for at a given moment, and (4) short text or audio explanations. Content editing can be done in professional video editing software (eg, Adobe Premiere or CapCut [Bytedance Ltd]). Educators design each content block to support a single outcome-activity pair, which helps maintain cognitive load at a manageable level and supports germane processing [15,20,21].

The content blocks for all targeted outcomes are assembled on the VR platform (eg, Spatial.io) to create a complete VR module for that procedure. For each major outcome, students first encounter a concise explanation and then watch an immersive 360-degree demonstration of the corresponding actions in the imaging room. Pop-up guides and labels draw attention to critical details, and students can pause or replay segments to review challenging steps. At selected points, brief prompts ask them to reflect on what was done to reinforce learning, in line with recommendations for structured briefing and debriefing in simulation-based education [4,8]. Subsequently, students participate in formative assessment and feedback, which helps them consolidate understanding, evaluate performance, and identify areas for improvement. The framework aims to make VR-based procedural teaching both accessible and scalable for RT educators.

### Contrast-Enhanced CT Brain Learning Module

**Figure 2.**
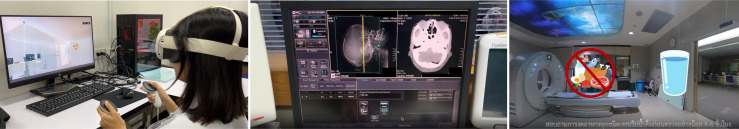
Learning environments in the virtual reality (VR) module for contrast-enhanced computed tomography (CT) brain examination training. (Left) A radiologic technology (RT) student using the head-mounted VR device (Meta Quest 3) during the 20-minute learning session. (Middle) CT console interface within the VR environment used to familiarize students with scan planning and parameter selection. (Right) CT room scene with instructional overlays highlighting equipment and workflow steps.

The VR module developed for this study focused on patient preparation and procedural workflow for a CECT brain examination (see Figure 2). The module was designed using the RTVR instructional design framework, starting from the learning outcome: students will be able to describe and sequence the key steps in safely preparing a patient for a CECT brain examination, including screening, positioning, contrast administration, and immediate postscan care.

**Figure 3.**
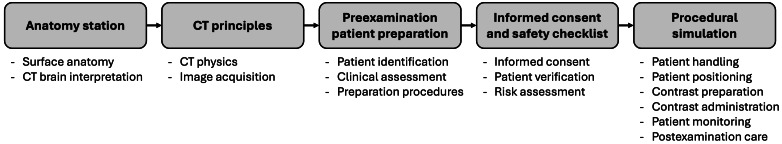
Structure of the virtual reality (VR) learning module for contrast-enhanced computed tomography (CECT) brain examination, organized into 5 instructional components: anatomy station, computed tomography (CT) principles, pre-examination patient preparation, informed consent and safety procedures, and procedural simulation.

The learning activities derived from this outcome included the following: a brief concept introduction on indications, risks, and safety checks; VR-based environment orientation in the CT suite; a step-by-step 360-degree demonstration of patient preparation before, during, and after the scan; and short reflection prompts that asked students to notice correct screening, positioning, contrast handling, and postexamination monitoring. The module was organized into 5 instructional components, as shown in Figure 3. The learning components were later decomposed into small VR content blocks. Components 1 to 4 consisted primarily of learning materials, including a 3D model of a CT machine with labeled equipment overlays; educational posters; signs; protocols; checklists; anatomy landmarks; and audio segments explaining principles, key concepts, procedures, and safety. Component 5 (Procedural Simulation) used 360-degree video captures for procedural demonstration, including prescan patient preparation, intravenous line and contrast preparation, positioning with correct scan range and table height, instructions during injection, and postscan observation. The 360-degree video captures were overlaid with equipment and position labels, and short text or audio segments explained what the radiologic technologist was doing at that moment. Although all 5 components were included in the module, components 1 to 4 were designed as brief preparatory learning materials, whereas most of the learning time was spent in component 5.

All immersive media were created using a standardized 360-degree video production pipeline. A dual-lens 360-degree camera (Insta360 X3) was mounted on a 1.2-m monopod positioned at the radiologic technologist’s eye height to approximate a first-person clinical viewpoint. Lighting conditions were controlled using the existing CT suite illumination. Each procedural phase was filmed as a discrete take to support later decomposition into content blocks. Ambient audio was captured using the camera’s built-in microphone. 360-degree captures were imported into video-editing software (Adobe Premiere Pro, version 24.2; Adobe Inc). Each clip was trimmed and segmented into content blocks. Instructional overlays and pop-up guides were added.

These content blocks are assembled in an immersive content authoring platform (Spatial, Spatial Systems Inc) to create a structured VR learning module in which learners progress through the 5 ordered components, experiencing each component in a head-tracked 360-degree environment. Within each component, participants could look freely around the 360-degree clinical environment; activate embedded labeled overlays to identify CT room equipment, patient positioning landmarks, contrast preparation materials, and key workflow locations; and trigger pop-up guides that appeared at predefined points in the scene. Participants could also pause and revisit content blocks. The primary mode of engagement was observation and guided attention rather than free manipulation of objects.

The VR experience followed a structured sequence. The session began with a prebrief in which the instructor stated the learning outcome, explained the module structure, and oriented students to VR use. Students then completed the VR activity using a head-mounted display, progressing through the CECT brain VR module. The session ended with a short, instructor-led debrief in which students reconstructed key steps and clarified uncertainties, followed by a multiple-choice knowledge test aligned with the learning outcome. The VR module content and the conventional learning materials were not modified after the study commenced.

The VR module is not publicly accessible because it contains voice recordings, 360-degree video recordings of identifiable individuals, and proprietary 3D assets. The module may be made available by the corresponding author upon reasonable request and subject to institutional data access procedures and the consent provided by the individuals involved.

### Trial Design

A randomized, controlled, open-label, parallel-group pretest–posttest design was used to evaluate the effectiveness of the CECT brain VR learning module. The study compared the VR module with a conventional document-based learning module. Before the study began, 3 subject matter experts independently reviewed the VR module to evaluate its content accuracy and technology acceptance. The study was registered in the Thai Clinical Trials Registry (TCTR20260309005) [22], where the study protocol is available. Registration was retrospectively completed on March 9, 2026, following study completion on February 23, 2025. No separate statistical analysis plan was preregistered. This manuscript was prepared in accordance with the CONSORT-EHEALTH (Consolidated Standards of Reporting Trials of Electronic and Mobile Health Applications and Online Telehealth) guidelines (Checklist 1) [23].

### Patient and Public Involvement

No patients or members of the public were involved in the design, conduct, or reporting of this study. The study population consisted of RT students who participated as research participants.

### Trial Setting and Participants

Participants were recruited through a posted notice in the Radiological Technology Program, Department of Radiology, Faculty of Medicine, Prince of Songkla University, Thailand. The notice described the study purpose, voluntary nature of participation, and the 2 possible learning conditions. Students who were interested provided written informed consent before enrollment.

The inclusion criteria were as follows: (1) undergraduate RT students enrolled in year 2 or year 3 of the Radiological Technology Program, Faculty of Medicine, Prince of Songkla University; (2) completion of the Diagnostic Radiology Equipment course; (3) not yet enrolled in the Special Diagnostic Radiologic Techniques II course; (4) no prior clinical training in diagnostic radiology practice; and (5) voluntary agreement to participate with written informed consent. The exclusion criteria were as follows: (1) a history of cybersickness, motion sickness, or vestibular disorders related to VR use and (2) development of dizziness, nausea, or discomfort during VR use that prevented continuation of participation. Prior VR experience was not an inclusion criterion. Prior VR experience or use was not formally assessed at baseline.

Thirty-six RT students (18 second-year and 18 third-year students) from the Radiological Technology Program, Department of Radiology, Faculty of Medicine, Prince of Songkla University, Thailand, participated in the study. Data collection was conducted between February 15 and February 23, 2025. No notable changes to VR hardware, the software platform, the VR module, or clinical learning infrastructure occurred during the study period. Regarding prior academic background, year-2 students had completed core RT courses in year 1, covering basic sciences, anatomy, physiology, and radiation physics, and year 2 coursework in diagnostic radiology instrumentation. Year-3 students had completed all year-1 and year-2 courses, as well as year-3 coursework in radiological techniques and modality-specific content. Students in both groups had not yet enrolled in the advanced diagnostic radiologic techniques course, which covers CT and CECT protocols, and had no prior clinical training in diagnostic radiology practice.

### Randomization

Participants were randomly allocated in a 1:1 ratio to either the VR intervention group or the conventional document-based control group using a stratified ballot draw, with separate ballots for year-2 and year-3 students to ensure equal strata in each group. Each participant drew their own ballot from an opaque container. After the participants were enrolled and provided consent, they were randomized by research assistants independent of outcome assessment. Blinding of participants was not possible because the 2 learning formats were clearly distinguishable. Data analysis was performed by JC and SC, who were blinded to individual participant identities. Both groups participated in self-directed learning activities under researcher monitoring, with no real-time instructor involvement during module use. Because participants were aware of their assigned group, expectation effects and differences in motivation between groups could not be completely excluded. No participants withdrew or were excluded after randomization.

### Interventions

The study proceeded through 3 phases. First, both groups completed an identical 20-item pretest (15 min). Next, during the intervention phase, the VR group completed the CECT brain VR module for 20 minutes using a head-mounted display (Meta Quest 3, Meta Platforms Inc). The control group studied chapters 10 to 14 of the standard curriculum textbook *Computed Tomography for Technologists* (Romans [24]) for a standardized 20 minutes, covering the same procedural content as the VR module. Simultaneously, physiological data were collected from VR participants before, during, and after media use to explore their responses during the immersive learning experience. Finally, both groups completed an identical 20-item posttest (15 min) to measure knowledge acquisition. Students in the VR group also provided usability and satisfaction ratings for the module. No reminders, prompts, or follow-up contacts were used, as this was a single-session study.

### Outcomes

The primary knowledge outcome was assessed using a 20-item multiple-choice questionnaire, which was aligned with the curriculum outcomes used in the VR design. The 20-item questionnaire is provided in Multimedia Appendix 1. Each item had 4 response options, including 1 correct answer, resulting in a total score range of 0 to 20. Items covered 7 content domains aligned with the CECT brain procedural workflow: (1) brain anatomy and imaging reference landmarks; (2) scan range, patient positioning, and isocenter setup; (3) contrast media selection based on renal function; (4) patient screening and premedication indications and timing; (5) contrast injection parameters; (6) adverse reactions and complication management; and (7) postscan patient care. The test was validated by 3 subject matter experts prior to use.

Six categories of quantitative measures were used in the evaluation. All outcomes were prespecified prior to data collection and were not changed after the study commenced. No qualitative feedback was collected from participants.

- Content validation: three subject matter experts evaluated the VR module. First, they assessed the content validity of the 20-item knowledge test using the content validity index (CVI) [25]. For each item, each expert rated its relevance as either relevant (score=1) or not relevant (score=0), and the item-level CVI (I-CVI) was calculated as the proportion of experts who rated that item as relevant. The scale-level (S-CVI) was then computed as the average of all I-CVI scores across the full test.
- Learning media appropriateness: a 10-item evaluation form was used to assess the learning media appropriateness of the VR module by 3 experts. The instrument measured content clarity, lesson sequencing, media presentation, audiovisual quality, and usability.
- Pretest and posttest: the 20-item multiple-choice knowledge test was applied for the pretest and posttest to assess the understanding of key procedural concepts and measure learning gains.
- Physiological measurements: for VR participants, systolic blood pressure (SBP) and diastolic blood pressure (DBP), and heart rate (HR) measurements were taken before (after the pretest), during (midway through the session), and after (following the posttest). They served as supplementary indicators of the learning experience during VR immersion, specifically to detect potential signs of physiological stress or discomfort that might be associated with VR use. These measurements were selected as noninvasive, readily obtainable indices of physiological arousal, consistent with their use in prior VR education research [21].
- Technology acceptance: a 12-item Technology Acceptance Model (TAM) instrument was adapted to evaluate the innovation acceptance of the VR module. The questionnaire included 3 constructs: perceived usefulness, perceived ease of use, and behavioral intention. Experts (n=3) and VR participants (n=18) assessed the module using the same 5-point Likert scale.
- Student satisfaction: VR participants completed a satisfaction survey after the posttest assessment. Both the expert content evaluation and the student satisfaction surveys used the same 10-item evaluation form. While experts evaluated learning media appropriateness, students evaluated satisfaction and perceived quality. The survey was designed to assess student perception of the VR module specifically rather than general satisfaction with instructional methods.

### Harms

Adverse events, cybersickness, and VR-related discomfort were monitored throughout the study session via direct researcher observation. Any participant who developed dizziness, nausea, or discomfort during VR use was excluded and the event recorded. No systematic cybersickness scale was administered. Monitoring was passive and based on participant self-report and researcher observation.

### Sample Size

We considered the primary effect of interest to be the difference in knowledge gains between the VR and control groups. A priori power analysis for a 2-sample comparison of gain scores (*α*=.05, 2-sided; power=0.80) indicated that 63 participants per group (126 total) would be required to detect a medium effect (Cohen *d*=0.50). Our actual sample (n=18 per group) therefore had adequate power to detect large effects (*d*>0.80) but may have been underpowered for medium effects. Expected attrition was not incorporated into the calculation because this was a single-session educational study conducted in a fixed student cohort, and dropout was expected to be minimal. No interim analyses or stopping guidelines were planned or conducted.

### Statistical Methods

Descriptive statistics were computed for all variables. Expert validation ratings were summarized using mean and SD values.

To evaluate knowledge acquisition and compare the efficacy of the instructional methods, a linear mixed model (LMM) was used as the primary analysis. The LMM was fitted with test (pretest vs posttest), group (VR vs control), and year (year 2 vs year 3) as fixed effects, along with all pairwise interactions (test × group, test × year, and year × group) and the 3-way interaction (test × year × group). A random intercept for each participant was included to account for the repeated measurements within individuals. Degrees of freedom were estimated using the Satterthwaite method. Model fit was evaluated using marginal *R*² and conditional *R*². The primary analysis followed an intention-to-treat approach. All 36 randomized participants completed both the pretest and posttest assessments with no dropouts, so the intention-to-treat population was identical to the per-protocol population, and no imputation was required.

For physiological variables, a 1-way repeated-measures ANOVA was performed with time (before, during, and after the learning session) as a within-subjects factor, separately for SBP, DBP, and HR. Post hoc pairwise comparisons between time points were conducted for each physiological measure using paired-samples *t* tests with Holm correction. Pearson correlation coefficients (*r*) were computed to evaluate the relationship between learning gains (posttest score − pretest score) and physiological changes across time points. Student satisfaction scores were analyzed descriptively. Statistical significance was set at *P<.*05. Statistical analyses were performed using Jamovi software (version 2.6; Jamovi Project) [26], R software (version 4.4; R Foundation for Statistical Computing) [27], and Python (version 3.12; Python Software Foundation).

### Ethical Considerations

This study was reviewed and approved by the Human Research Ethics Committee of the Faculty of Medicine, Prince of Songkla University (approval REC.67-461-7-2; approved November 8, 2024). All procedures were conducted in accordance with the 1964 Declaration of Helsinki and its later amendments, ensuring the protection, rights, and safety of all participants. Written informed consent was obtained from all participants prior to enrollment. Participants were informed of the study purpose, procedures, voluntary nature of participation, and their right to withdraw at any time without consequence. All study data were collected and stored in a deidentified form. No personally identifiable information was retained in the research dataset. Data are held securely in accordance with the institutional policy of the Faculty of Medicine, Prince of Songkla University.

Participants received no financial compensation for participation. Participation was entirely voluntary. No images in this article or supplementary materials contain identifiable individuals. Any figures depicting participants show only VR equipment or aggregate data.

## Results

### Baseline Characteristics

**Figure 4.**
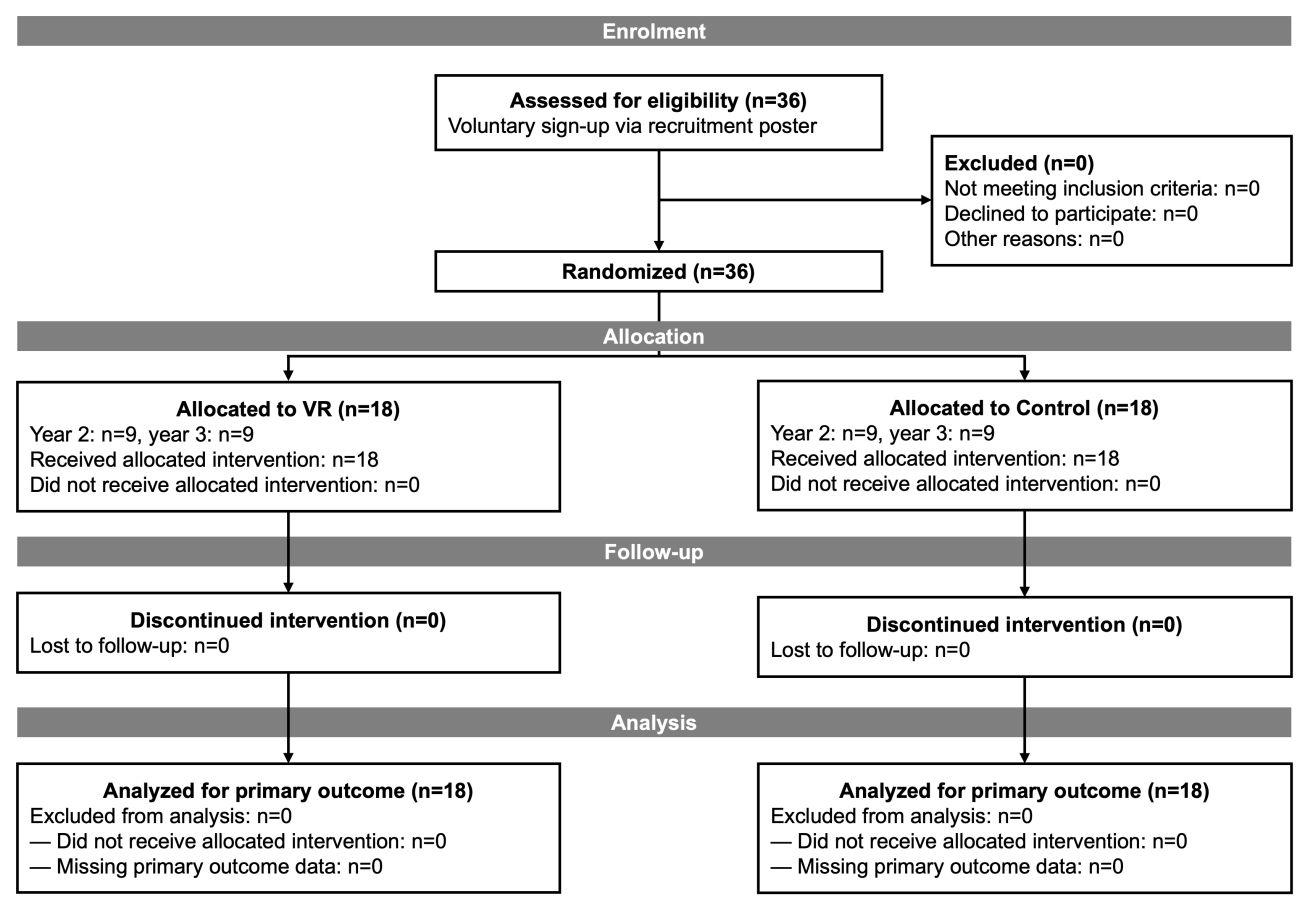
CONSORT participant flow diagram. VR: virtual reality.

Baseline characteristics by group are presented in Table 1. Academic year was balanced across groups by design, with 9 second-year and 9 third-year students in each group. Age was not collected as part of the study protocol. All participants in both the VR and control groups completed the full 20-minute learning activities in a single uninterrupted session, and no participant required early termination. The participant flow is shown in Figure 4.

**Table 1.** Baseline characteristics of participants by intervention group (N=36)^a^.

Characteristic	VR^b^ group (n=18)	Control group (n=18)
Academic year, n (%)		
Year 2	9 (50.0)	9 (50.0)
Year 3	9 (50.0)	9 (50.0)
Sex, n (%)		
Male	5 (27.8)	7 (38.9)
Female	13 (72.2)	11 (61.1)

a Age was not collected as part of the study protocol.

b VR: virtual reality.

### Content Validity

Three experts (JC, NI, and SKM) evaluated the content validity of the VR module using the CVI across the 20-item, multiple-choice knowledge test. All experts were radiologic technologists and faculty members with more than 10 years of experience in RT practice and held a master’s degree or higher in medical physics or RT. Every item received a relevance score of 1 from all 3 experts. This yielded both an I-CVI and a S-CVI of 1.00. These results confirmed that the test was valid and suitable for the study. We acknowledge that all 3 experts are coauthors, and JC is also a lead developer of the module, which may introduce evaluator bias. To mitigate this, NI and SKM provided independent evaluations without prior discussion.

### Learning Media Appropriateness

The same subject matter experts (JC, NI, and SKM) evaluated the appropriateness of the learning media across 10 dimensions (see Table 2). The assessment had a high overall mean score of 4.70 (SD 0.38). Half of the evaluation items, specifically those related to understanding the procedure, engagement, visual clarity, and audio quality, received perfect scores (mean 5.00, SD 0.00). These results confirmed that the VR module was suitable for the study.

**Table 2.** Evaluation of learning media appropriateness of the virtual reality module for contrast-enhanced computed tomography (CECT) brain examination training, based on ratings by 3 subject matter experts (radiologic technology faculty, >10 years of experience) using a 10-item 5-point Likert scale (5=highest, 1=lowest)^a^.

Evaluation item	Score, mean (SD)
The learning media helps in understanding the process of patient preparation and the steps of the CECT brain examination.	5.00 (0.00)
The sequence of lesson presentation is appropriate.	4.67 (0.58)
The learning media has an engaging format.	5.00 (0.00)
The learning media provides clear and sharp images.	5.00 (0.00)
The learning media is presented in a continuous manner.	4.33 (0.58)
The learning media is enjoyable.	5.00 (0.00)
The learning media can be used for lesson review.	4.67 (0.58)
The learning media is convenient to use.	4.67 (0.58)
The learning media has clear and audible sound.	5.00 (0.00)
The learning media includes clear descriptions.	3.67 (1.53)
Overall average	4.70 (0.38)

a 95% CIs are not reported for n=3 due to insufficient precision.

### Knowledge Gains

Descriptive statistics for all subgroups are presented in Table 3. For year-2 students using VR, the mean score increased from 5.89 (SD 2.15) on the pretest assessment to 15.78 (SD 2.33) on the posttest assessment. The mean score of year-3 students using VR improved from 10.78 (SD 1.56) to 17.67 (SD 1.58). In the control group, the mean score of year-2 students improved from 7.33 (SD 3.04) to 16.89 (SD 1.69), and that of year-3 students improved from 10.89 (SD 1.83) to 18.00 (SD 1.12).

**Table 3.** Pretest and posttest knowledge scores (20-item multiple-choice test; maximum score 20) by instructional group and academic year for 36 radiologic technology students.

Group, year, and test	Score, mean (SD)	95% CI
VR^a^		
Y2^b^		
Pretest	5.89 (2.15)	4.24-7.54
Posttest	15.78 (2.33)	13.98-17.57
Y3^c^		
Pretest	10.78 (1.56)	9.58-11.98
Posttest	17.67 (1.58)	16.45-18.88
Control		
Y2		
Pretest	7.33 (3.04)	5.00-9.67
Posttest	16.89 (1.69)	15.59-18.19
Y3		
Pretest	10.89 (1.83)	9.48-12.30
Posttest	18.00 (1.12)	17.14-18.86

a VR: virtual reality.

b Y2: year 2.

c Y3: year 3.

**Figure 5.**
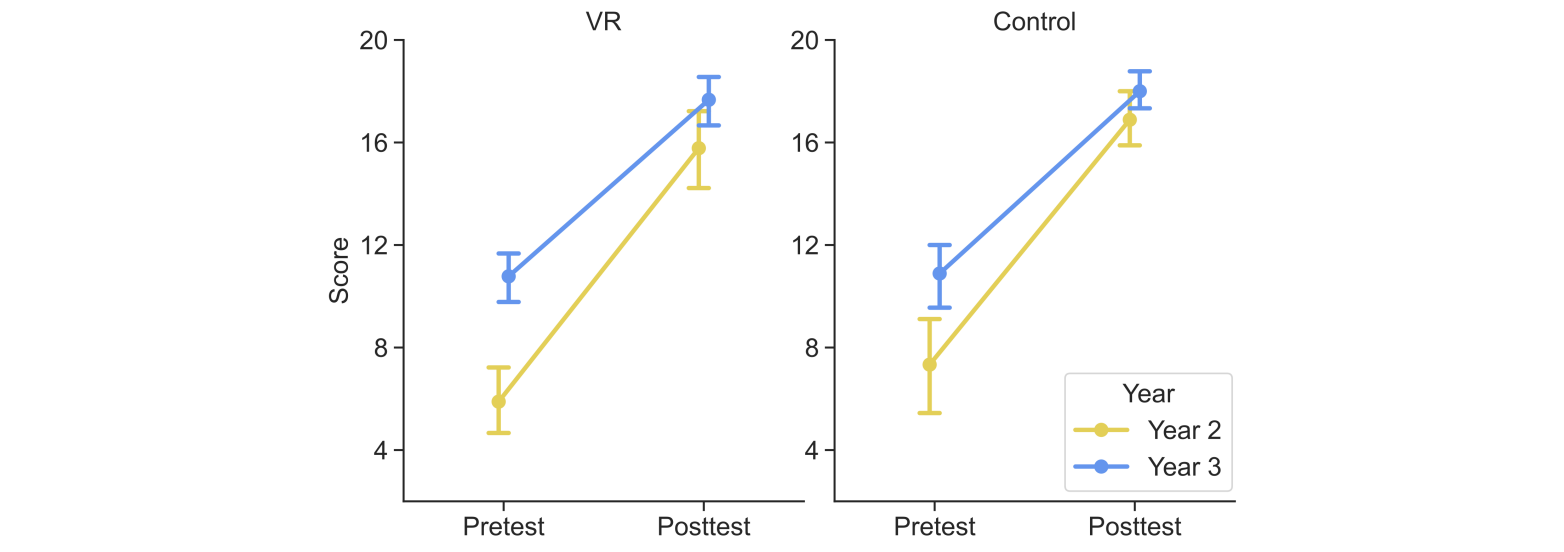
Pretest and posttest knowledge scores on a 20-item multiple-choice test assessing declarative knowledge of contrast-enhanced computed tomography brain procedural workflow, by instructional group (virtual reality [VR] vs control) and academic year (year 2 vs year 3), of 36 radiologic technology students. Error bars represent 95% CIs.

The LMM results are presented in Table 4. The model explained 83.8% of variance in scores through fixed effects (marginal *R*²=0.838) and 90.7% of variance in scores when the random participant intercept was included (conditional *R*²=0.907; intraclass correlation coefficient=0.429). The statistical analysis yielded the following results:

- Learning effectiveness: a significant main effect of test confirmed substantial knowledge acquisition in both instructional methods (unstandardized regression coefficient β=8.361, SE 0.355, 95% CI 7.652-9.070; *t*_32_=23.58, *P*<.001).
- Comparison of instructional methods: the test × group interaction was not significant (unstandardized regression coefficient β=0.056, SE 0.709, 95% CI −1.360 to 1.473; *t*_32_=0.08, *P*=.94), indicating that no statistically significant difference in knowledge gains was detected between the 2 instructional methods. The 95% CI for this interaction was narrow and centered near zero, suggesting that any true difference in knowledge gain between the 2 methods is unlikely to exceed approximately 1.5 points on a 20-point scale. However, given that the study was powered only for large effects, a medium-sized difference cannot be ruled out. The between-subjects main effect of group was also nonsignificant (unstandardized regression coefficient β=−0.750, SE 0.561, 95% CI −1.870 to 0.372, *t*_32_=−1.34, *P*=.19).
- Impact of academic year: a significant test × year interaction (unstandardized regression coefficient β=−2.722, SE 0.709, 95% CI −4.140 to −1.304; *t*_32_=−3.84, *P*<.001) and a significant main effect of year (unstandardized regression coefficient β=2.861, SE 0.561, 95% CI 1.740-3.983; *t*_32_=5.10, *P*<.001) were identified. While both cohorts improved, year-2 students showed greater pretest to posttest gains than year-3 students, consistent with lower baseline knowledge among year-2 students (see Figure 5).
- Interaction of all factors: the 3-way interaction between test, year, and group was not significant (unstandardized regression coefficient β=−0.556, SE 1.419, 95% CI −3.391 to 2.280; *t*_32_=−0.39, *P*=.70). The relationship between academic year and learning gains were consistent across both methods.

**Table 4.** Fixed effects estimates from the LMM^a^ for 20-item multiple-choice knowledge test scores, with test (pretest vs posttest), group (VR^e^ vs control), and year (year 2 vs year 3) as fixed effects and a random intercept per participant (data from 36 radiologic technology students).

Effect	β^c^	SE	95% CI	*t* test (*df*)	*P* value	
Intercept	12.903	0.281	12.342 to 13.464	45.99 (32)	<.001	
Test (posttest – pretest)	8.361	0.355	7.652 to 9.070	23.58 (32)	<.001	
Year (Y3 – Y2)	2.861	0.561	1.740 to 3.983	5.10 (32)	<.001	
Group (VR – control)	−0.750	0.561	−1.870 to 0.372	−1.34 (32)	.19	
Test × year	−2.722	0.709	−4.140 to −1.304	−3.84 (32)	<.001	
Test × group	0.056	0.709	−1.360 to 1.473	0.08 (32)	.94	
Year × group	1.056	1.122	−1.188 to 3.300	0.94 (32)	.35	
Test × year × group	−0.556	1.419	−3.391 to 2.280	−0.39 (32)	.70	

a LLM: linear mixed model.

b VR: virtual reality.

c β: unstandardized regression coefficient.

### Physiological Measures

**Figure 6.**
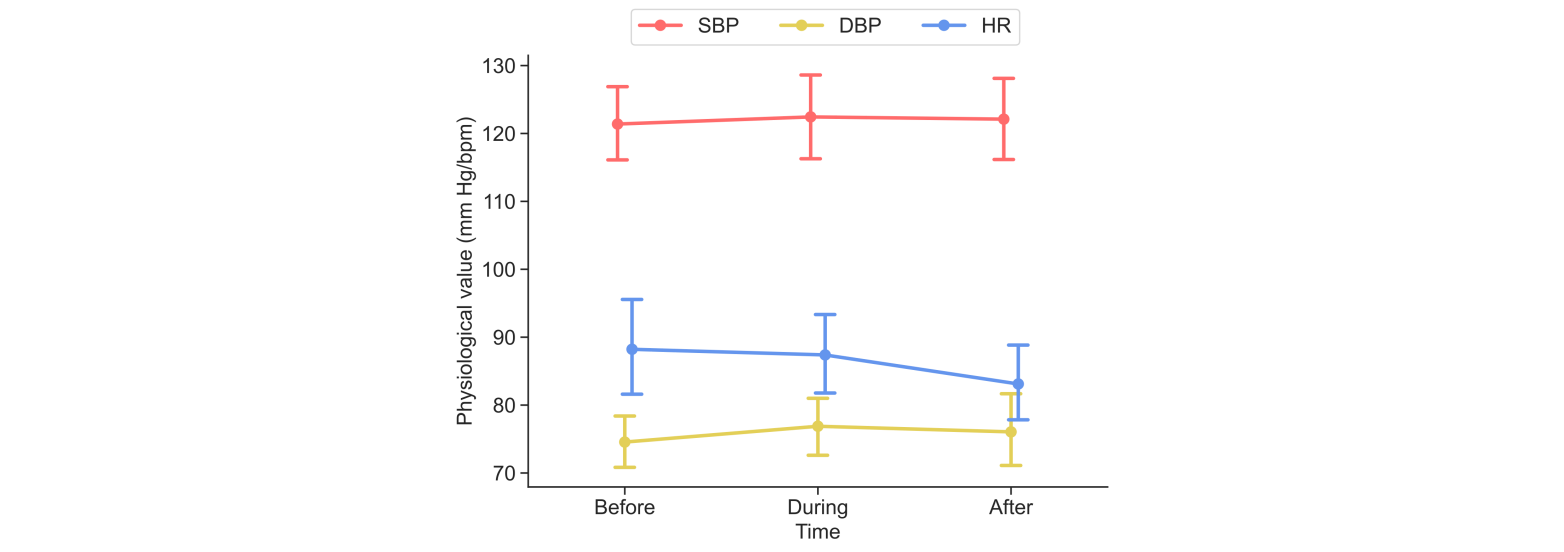
Physiological responses (SBP [mm Hg], DBP [mm Hg], and HR [bpm]), measured at 3 time points (before, during, and after the 20-minute VR session) in the VR group (n=18 students). Plots represent mean and 95% CI values. bpm: beats per minute; DBP: diastolic blood pressure; HR: heart rate; SBP: systolic blood pressure; VR: virtual reality.

Physiological responses (SBP, DBP, and HR) were recorded across 3 time points in the VR group (see Figure 6). As shown in Table 5, repeated-measures ANOVA revealed no significant changes in SBP (*F*_2_*_,_*_34_=0.144, *P*=.87) or DBP (*F*_2_*_,_*_34_=0.597, *P*=.56) across the session. In contrast, a significant main effect of time was observed for HR (*F*_2_*_,_*_34_=5.52, *P*=.008, *η_p_*^2^=.245), with values decreasing progressively from before the learning session to after the learning session. Paired comparisons with Holm correction (see Table 6) indicated that HR was significantly lower after the learning session than both before (mean difference [MD]=−5.11 bpm, 95% CI −9.20 to −1.02) and during (MD=−4.28 bpm, 95% CI −7.47 to −1.08) the learning session, whereas no pairwise differences were observed for SBP or DBP.

**Table 5.** Physiological measures (SBP^a^, DBP^b^, and HR)^c^ across 3 time points (before, during, and after the 20-minute learning session) in the virtual reality group.

Vital sign	Time point, mean (SD; 95% CI)			ANOVA^d^ results		
	Before	During	After	*F* test (*df*)	*P* value	$\eta_{p}^{2}$
SBP (mm Hg)	121.4 (12.3; 115.3-127.5)	122.4 (13.2; 115.9-129.0)	122.1 (14.4; 115.0-129.3)	0.144 (2, 34)	.87	.008
DBP (mm Hg)	74.6 (8.5; 70.3-78.8)	76.9 (9.2; 72.3-81.5)	76.1 (11.7; 70.3-81.9)	0.597 (2, 34)	.56	.034
HR (bpm)^e^	88.2 (15.5; 80.5-95.9)	87.4 (13.6; 80.6-94.2)	83.1 (12.1; 77.1-89.1)	5.52 (2, 34)	.008	.245

a SBP: systolic blood pressure (mm Hg).

b DBP: diastolic blood pressure (mm Hg).

c HR: heart rate (bpm).

d One-way repeated-measures ANOVA results are shown.

e bpm: beats per minute.

**Table 6.** Comparisons of physiological measures between time points (before, during, and after the 20-minute training session) in the virtual reality group using paired-samples *t* tests with Holm correction.

Change	SBP^a^ (mm Hg)			DBP^b^ (mm Hg)			HR^c^ (bpm^d^)		
	*t* test (*df*)	MD^e^ (95% CI)	*P* value	*t* test (*df*)	MD (95% CI)	*P* value	*t* test (*df*)	MD (95% CI)	*P* value
During – before	0.634 (17)	1.06 (−2.46 to 4.57)	>.99	1.867 (17)	2.33 (−0.30 to 4.97)	.24	−0.573 (17)	−0.83 (−3.90 to 2.23)	.57
After – before	0.362 (17)	0.72 (−3.48 to 4.93)	>.99	0.631 (17)	1.50 (−3.51 to 6.51)	>.99	−2.634 (17)	−5.11 (−9.20 to −1.02)	.04
After – during	−0.144 (17)	−0.33 (−5.23 to 4.56)	>.99	−0.318 (17)	−0.83 (−6.36 to 4.69)	>.99	−2.825 (17)	−4.28 (−7.47 to −1.08)	.04

a SBP: systolic blood pressure.

b DBP: diastolic blood pressure.

c HR: heart rate.

d bpm: beats per minute.

e MD: mean difference.

Table 7 summarizes the Pearson correlations between learning gains and physiological changes. A moderate negative correlation emerged between the change in SBP between before the learning session and during the session and test score improvement (*r*=−0.50, 95% CI −0.78 to −0.04, *P*=.04). Greater SBP during immersion was associated with smaller learning gains. All remaining correlations for SBP, DBP, and HR across the 3 intervals were small and nonsignificant, with 95% CIs that included zero in all cases (see Table 7).

**Table 7.** Pearson correlations between knowledge gain (posttest score – pretest score) and physiological changes across time-point intervals, in the virtual reality group.

Change	SBP^a^ (mm Hg)		DBP^b^ (mm Hg)		HR^c^ (bpm^d^)	
	*r^e^* (95% CI)	*P* value	*r* (95% CI)	*P* value	*r* (95% CI)	*P* value
During – before	−0.50 (−0.78 to −0.04)	.04	0.17 (−0.33 to 0.59)	.51	−0.04 (−0.50 to 0.43)	.87
After – before	−0.08 (−0.52 to 0.41)	.76	0.42 (−0.05 to 0.74)	.08	−0.01 (−0.47 to 0.46)	.97
After – during	0.29 (−0.21 to 0.67)	.24	0.31 (−0.19 to 0.68)	.22	0.03 (−0.44 to 0.49)	.91

a SBP: systolic blood pressure.

b DBP: diastolic blood pressure .

c HR: heart rate.

d bpm: beats per minute.

e *r*: Pearson correlation coefficient.

These physiological findings should be interpreted as exploratory process measures of learner comfort during VR exposure. Decreasing HR and stable SBP and DBP suggest that the VR session was generally well tolerated. They do not constitute evidence of educational effectiveness.

### Technology Acceptance

The technology acceptance of the VR module was evaluated by 3 experts and 18 VR participants (see Table 8). The expert group reported a high overall acceptance (mean 4.86, SD 0.17), with maximal scores (mean 5.00, SD 0.00) reported for multimedia presentation, assessment tools, and interactivity. Similarly, the student group reported strong acceptance with an overall mean of 4.80 (SD 0.23, 95% CI 4.69-4.92), with the clarity of symbols and images rated particularly high (mean 5.00, SD 0.00).

**Table 8.** Results of the Technology Acceptance Model (TAM) evaluation by experts (n=3) and students (n=18).^a^

Evaluation item	Experts	Students
	Score, mean (SD)	Score, mean (SD; 95% CI)
Perceived usefulness (PU)		
1. The VR^b^ content effectively presents the procedural steps for CECT^c^ training.	4.67 (0.58)	4.78 (0.43; 4.57-4.99)
2. The presentation of images and multimedia enhances understanding of the CECT process.	5.00 (0.00)	4.83 (0.38; 4.64-5.02)
3. The VR module provides clear and useful simulated assessment tools.	5.00 (0.00)	4.83 (0.38; 4.64-5.02)
Overall PU	4.89 (0.19)	4.81 (0.31; 4.66-4.97)
Perceived ease of use (PEOU)		
4. Menu commands are easy to navigate.	4.67 (0.58)	4.72 (0.46; 4.49-4.95)
5. Layout and menu placement are intuitive.	5.00 (0.00)	4.89 (0.32; 4.73-5.05)
6. Font type, size, and color are appropriate.	4.67 (0.58)	4.78 (0.43; 4.57-4.99)
7. Explanatory text clearly conveys key content.	5.00 (0.00)	4.72 (0.46; 4.49-4.95)
8. Symbols and images effectively communicate information.	4.67 (0.58)	5.00 (0.00; 5.00-5.00)
9. The VR training is straightforward to use without unnecessary complexity.	5.00 (0.00)	4.83 (0.51; 4.58-5.09)
10. Interactive features respond appropriately and support learning.	5.00 (0.00)	4.78 (0.43; 4.57-4.99)
Overall PEOU	4.86 (0.14)	4.82 (0.23; 4.70-4.93)
Behavioral intention (BI)		
11. I anticipate to use this VR system for practice or training activities.	4.67 (0.58)	4.67 (0.49; 4.43-4.91)
12. I would recommend this VR training system to other students or educators.	5.00 (0.00)	4.78 (0.55; 4.51-5.05)
Overall BI	4.83 (0.29)	4.72 (0.39; 4.53-4.92)
Overall TAM (PU, PEOU, BI)	4.86 (0.17)	4.80 (0.23; 4.69-4.92)

a 95% CIs are reported for student ratings (n=18) only. Expert ratings (n=3) were insufficient for meaningful interval estimation.

b VR: virtual reality.

c CECT: contrast-enhanced computed tomography.

### Student Satisfaction Surveys

Eighteen VR participants evaluated their satisfaction with the VR module (see Table 9). Students reported a very high level of overall satisfaction (mean 4.87, SD 0.29, 95% CI 4.73-5.01). Specifically, the clarity of the descriptions received a perfect rating (mean 5.00, SD 0.00, 95% CI 5.00-5.00). The appropriateness of the lesson sequence, engagement, continuity, enjoyment, and audio quality all received exceptionally high ratings (mean 4.94, SD 0.24, 95% CI 4.83-5.06).

**Table 9.** Results of the satisfaction surveys by VR^a^ participants (n=18).

Evaluation item	Score^b^, mean (SD; 95% CI)
1. The learning media helps in understanding the process of patient preparation and the steps of the CECT^c^ brain examination.	4.78 (0.43; 4.57-4.99)
2. The sequence of lesson presentation is appropriate.	4.94 (0.24; 4.83-5.06)
3. The learning media has an engaging format.	4.94 (0.24; 4.83-5.06)
4. The learning media provides clear and sharp images.	4.67 (0.49; 4.43-4.91)
5. The learning media is presented in a continuous manner.	4.94 (0.24; 4.83-5.06)
6. The learning media is enjoyable.	4.94 (0.24; 4.83-5.06)
7. The learning media can be used for lesson review.	4.89 (0.32; 4.73-5.05)
8. The learning media is convenient to use.	4.67 (0.49; 4.43-4.91)
9. The learning media has clear and audible sound.	4.94 (0.24; 4.83-5.06)
10. The learning media includes clear descriptions.	5.00 (0.00; 5.00-5.00)
Overall average	4.87 (0.29; 4.73-5.01)

a VR: virtual reality.

b Evaluation based on a 5-point Likert scale (5=highest, 1=lowest).

c CECT: contrast-enhanced computed tomography.

No adverse events, cybersickness incidents, or VR-related discomfort requiring discontinuation were observed during the study. No technical failures, equipment malfunctions, or data breaches occurred during data collection.

## Discussion

### Principal Findings

The primary aim of this study was to determine whether the RTVR framework, implemented as a CECT brain imaging module, can support declarative knowledge acquisition in RT students. Both the VR and conventional instruction groups showed substantial pretest to posttest knowledge gains, with no statistically significant difference in gain scores between the 2 groups. Year-2 students demonstrated greater improvement than year-3 students, consistent with their lower baseline knowledge of the CECT brain imaging procedure. Expert validation rated the module as suitable and appropriate for the target learning outcomes, and VR participants reported high technology acceptance and satisfaction. Physiological monitoring indicated that the VR session was well tolerated, with a progressive decrease in HR and SBP across the session.

Our primary outcome was declarative knowledge, assessed through a multiple-choice test. The findings therefore show that the VR module can deliver knowledge-focused procedural content effectively in this context. The results should not be interpreted as evidence of improved procedural skill, psychomotor ability, or clinical performance.

### Efficacy of the RTVR Framework

The primary analysis indicated no statistically significant difference in knowledge gains between the VR and conventional instruction groups. The test × group interaction was also not significant. Because conventional instruction produced similar gains, we cannot conclude that the immersive elements of the VR experience were responsible for the observed knowledge improvement. The VR module placed students in the clinical context without compromising knowledge acquisition. In practical terms, these findings indicate that the VR module can be used for knowledge-focused procedural teaching with learning outcomes comparable to those of conventional document-based instruction in this context.

This result is consistent with previous work indicating that, although VR often improves procedural performance, its advantage in declarative knowledge testing is typically comparable to text-based learning [21]. The concern that “seductive details” might distract learners was not supported in this study [28]. Instead, the RTVR framework appears to have effectively incorporated the principles of situated cognition [8,9], immersing learners in the clinical environment without compromising theoretical rigor. While O’Connor et al [16] positioned VR primarily as an augmentation to clinical skills laboratories and Taylor et al [14] emphasized the need for strong pedagogical grounding, our findings add to this literature by showing that a framework-driven, procedure-focused VR module can support knowledge-level learning alongside conventional instruction.

### Bridging the Experience Gap

The significant interaction between test and year showed that year-2 students, who had limited clinical exposure, demonstrated larger knowledge gains than year-3 students across the study. This pattern is consistent with the lower baseline knowledge of the year-2 cohort and their greater room for improvement on the posttest. At the same time, the nonsignificant test × year × group interaction indicates that this pattern was similar across instructional methods. The result therefore suggests that learner seniority shaped knowledge gain, rather than indicating a differential benefit of VR for one year level over the other. In practical terms, the VR module appears suitable for learners at different stages of training.

This finding aligns with Vu et al [19], who demonstrated that 360-degree virtual tours reduce anxiety and increase familiarity before clinical placement. Similarly, the VR module allowed younger learners to visualize workflows they had not yet encountered, providing environmental cues absent from textbooks. For more experienced learners, the module served as a meaningful reinforcement tool. These observations suggest that 360-degree VR may be particularly useful for early-stage learners who are beginning to build mental models of clinical workflow [20].

### Physiological Correlations and Learning Experience

One contribution of this study is the integration of physiological measures during VR immersion. Across the 3 time points, HR showed a significant main effect of time, with values decreasing from the beginning to the end of the session, whereas SBP and DBP remained stable. This pattern suggests that students remained physiologically settled during the VR activity, tolerated the activity well, and were, if anything, more relaxed by the end of the learning sequence. This indicates that the VR environment was comfortable and conducive to focused engagement rather than stress. The correlation analysis further supported this interpretation. A moderate negative association was observed between the change in SBP from before to during the learning session and learning gains, consistent with the idea that lower physiological arousal may be associated with stronger knowledge acquisition [29], in which moderate physiological arousal supports learning but elevated arousal may impair it. Other correlations were small and nonsignificant. This suggests that most physiological fluctuations were not strongly linked to learning performance. However, given the small sample size, these findings should be considered exploratory.

Taken together, these physiological results do not suggest pronounced technostress during VR use and are consistent with students remaining comfortable while engaging with the module. This is consistent with Taylor et al [14], who emphasized that VR solutions often fail due to insufficient pedagogical structure, and suggests that the segmented content block design of the RTVR framework may have contributed to a comfortable learning experience.

### Clinical and Educational Implications

Our findings suggest that the optimal approach is not choosing between VR and traditional methods but integrating both. For curriculum design, conventional materials (documents or lectures) can efficiently deliver foundational knowledge, followed by VR as a “virtual practicum” to apply concepts in a safe, realistic environment and build procedural confidence. This blended approach aligns with recommendations from Taylor et al [14], who emphasized that VR is most effective when embedded within structured learning cycles that include both preparation and debriefing. To bridge the theory-practice gap, VR can be introduced early in the curriculum to give junior students a tangible sense of the clinical environment before placement and used later as a refresher before senior students begin clinical rotations [19].

### Addressing VR Training Limitations

Prior research has highlighted limitations of VR, particularly its inability to replicate tactile skills such as palpation or patient communication [18]. This study, which focused on the procedural workflow of CECT brain scans, did not attempt to simulate haptic feedback. We acknowledge that VR cannot replace the physical experience of positioning a real patient. However, the RTVR framework mitigates this limitation by using a 360-degree video of real technologists performing authentic interactions rather than computer-generated avatars. This approach preserves realism.

We also acknowledge that our primary mode of engagement was guided observation rather than object manipulation, which is an inherent constraint of the 360-degree video format. This substantially limits the degree of interactivity available to learners. Participants could not manipulate equipment, reposition virtual patients, or make procedural decisions within the environment. Although our approach may maximize environmental realism and reduce content production costs, it does not provide the same level of active participation as more fully interactive VR environments.

### Study Limitations

This study is subject to the following limitations. First, the study was conducted at a single academic institution with a small sample size. With 18 participants per group, the study had sufficient power to detect large between-group effects, whereas smaller effects may not have been detectable. This may reduce the generalizability to other educational contexts. Second, the study relied on a 20-item multiple-choice test as the primary measure of learning outcomes. This design captured short-term declarative knowledge but did not evaluate procedural performance, psychomotor skills, or transfer to clinical practice. In addition, high posttest scores across all subgroups suggest a possible ceiling effect, which may have reduced the sensitivity of the multiple-choice test to detect smaller between-group differences after the intervention. Third, the short, single-session intervention design does not allow for conclusions on long-term knowledge retention, mental workload, or the effects of repeated practice. Next, we did not monitor physiological signs of cybersickness or use a validated self-report measure, which could have provided valuable data on its prevalence and impact. Furthermore, the study population consisted of Thai RT students at a single institution with no prior clinical radiology training, which may limit generalizability to programs with different curricula, VR literacy levels, or student backgrounds. Finally, physiological monitoring and satisfaction surveys were administered only to VR participants. This does not allow direct comparison of learner experience across instructional methods.

### Scalability and Future Directions

The study also addresses the scalability concerns outlined by Gårdling et al [15] and Taylor et al [14], who noted that high costs and technical demands limit VR adoption. The RTVR framework uses 360-degree video capture and off-the-shelf immersive content authoring platforms. This reduces the need for custom 3D modeling and specialized programming. This lowers the barriers to VR module creation, as new scenarios can be captured in real clinical environments with standard recording equipment and deployed with minimal technical overhead. This offers a significantly more feasible alternative to high-end VR systems such as HTC Vive used in earlier studies [16].

Because only knowledge-based outcomes were measured, future studies should evaluate whether VR-supported learning transfers to actual procedural performance using validated skill checklists or clinical assessments. Incorporating performance-based assessments, such as the rubric method proposed by Kato et al [18], would allow researchers to examine whether the spatial realism provided by VR translates into improved psychomotor performance in a physical CT suite.

The study did not include an intermediate comparator, such as a 2D video condition. It remains unclear whether the immersive format itself contributed meaningfully to learning beyond what a flat-screen video could achieve. Future studies should compare immersive 360-degree VR, nonimmersive 2D video, and conventional materials to isolate the specific contribution of immersion and to determine whether the additional cost and hardware requirements of VR are justified for knowledge-level outcomes.

The study measured outcomes immediately after the intervention, which is common in VR education research but does not allow conclusions on how well gains are sustained over time. A recent randomized controlled trial by Lindner et al [30] showed that VR-based simulation yielded higher delayed retention scores than video-based learning. However, Santilli et al [31] noted in a systematic review that only 4 of 71 VR education studies included a longitudinal retention assessment, which highlights a gap in the field. Future studies should include a delayed retention test to determine whether the knowledge gains observed here are maintained and whether VR-based learning confers any advantage over document-based or video-based instruction in long-term retention.

In this trial, VR sessions were preceded by a structured instructor-led prebrief and followed by a debrief, which are integral components of the RTVR framework. In routine deployment, the absence of trained facilitation or time constraints may prevent these elements, potentially reducing learning gains.

### Contributions and Implications

This study makes contributions to the RT education literature. First, it introduces the RTVR framework as a structured, scalable approach to immersive procedural content creation that does not require custom 3D environments or specialist technical skills. New modules can be built using existing clinical equipment, established institutional protocols, and real procedural experiences. Second, unlike prior VR studies in radiography that relied primarily on computer-generated environments [16-18], the RTVR framework uses a real 360-degree video of authentic clinical settings, providing the situated environmental cues that support contextual learning [8,9] while maintaining content authenticity. Third, this study pairs a randomized controlled trial design with physiological monitoring during VR immersion, offering a complementary indicator of learner experience alongside knowledge outcomes. Together, these contributions suggest that VR-based procedural training, when grounded in a systematic instructional design framework, is a feasible and evidence-informed supplement to existing RT curricula, supporting early procedural familiarity and helping bridge the theory-practice gap for early-stage learners.

### Conclusion

This study introduced and evaluated the RTVR framework to deliver a CECT brain imaging module. Experts validated the framework as suitable. Students reported high technology acceptance and satisfaction. Declarative knowledge increased from pretest to posttest, with no statistically significant difference in knowledge gain between the VR and control groups, while year-2 students showed larger gains than year-3 students across both instructional conditions. Physiological monitoring suggested that the VR session was generally well tolerated. Students praised the VR module for clarity, engagement, and realism. While VR cannot fully replicate tactile skills or patient communication, the RTVR framework reduces logistical barriers to VR content creation by using a 360-degree video instead of bespoke 3D models. Overall, the RTVR framework provides a safe, repeatable immersive learning environment and can be integrated with conventional instruction, with foundational knowledge delivered through traditional methods and reinforced through immersive experiences.

## Supplementary material

10.2196/88735 Multimedia Appendix 1 The 20-item pretest and posttest questionnaire (English translation).

10.2196/88735 Checklist 1 CONSORT-EHEALTH (Consolidated Standards of Reporting Trials of Electronic and Mobile Health Applications and Online Telehealth) checklist.

## Abbreviations

CECT: contrast-enhanced computed tomography
CONSORT-EHEALTH: Consolidated Standards of Reporting Trials of Electronic and Mobile Health Applications and Online Telehealth
CT: computed tomography
CVI: content validity index
DBP: diastolic blood pressure
HR: heart rate
I-CVI: item-level content validity index
LMM: linear mixed model
RT: radiologic technology
RTVR: radiologic technology virtual reality
S-CVI: scale-level content validity index
SBP: systolic blood pressure
VR: virtual reality
