# Supplementary material for: Virtual Reality–Based Training in Radiologic Technology for Contrast-Enhanced Computed Tomography Brain Imaging: Randomized Controlled Trial
Source: JMIR Form Res. 2026 May 26;10:e88735. doi: 10.2196/88735 (PMC13211598; doi:10.2196/88735)
Supplement: Multimedia Appendix 1 [file formative-v10-e88735-s001.pdf]

20-item Pre-test and Post-test Questionnaire for Knowledge Assessment

Item	Question	Answer
**1**	The line appearing in this image is called?<br>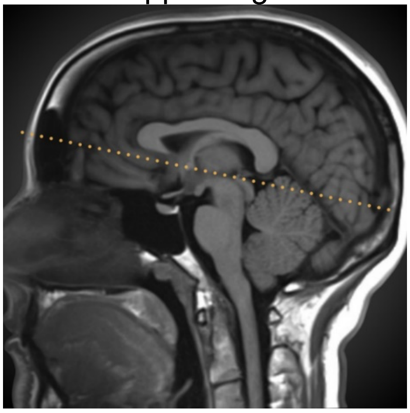<br>A. AC-PC line<br>B. Chamberlain's line<br>C. McGregor's line<br>D. Orbitomeatal line	**A**
**2**	The scan range for a CT brain corresponds to which option?<br>A. C1 – vertex of the skull<br>B. C3 – vertex of the skull<br>C. Base of the skull – EAM<br>D. Base of the skull – vertex of the skull	**D**
**3**	For patients with a GFR lower than 45 ml/min/1.73 m^2, which type of contrast media should be used?<br>A. Iso-osmolar<br>B. low-osmolar<br>C. high-osmolar<br>D. Both a and b are correct	**A**
**4**	Which group of patients should receive pre-medication before contrast media injection?<br>A. Hypertension patients<br>B. Severe heart disease patients<br>C. Cataract patients<br>D. Bladder stone patients	**B**
**5**	Which statement is correct regarding patient care after a CT brain with contrast?<br>A. Allow the patient to go home immediately<br>B. Restrict water intake for 24 hours after<br>C. Patient can eat normally<br>D. None are correct	**C**

Item	Question	Answer
**6**	For patients with a GFR higher than 45 ml/min/1.73 m^2, which type of contrast media should be used?<br>A. Iso-osmolar<br>B. low-osmolar<br>C. high-osmolar<br>D. Any type can be used	**D**
**7**	If the patient is uncooperative during examination, what should the radiologic technologist do?<br>A. Consult an anesthesiologist<br>B. Send the patient home and reschedule<br>C. Proceed with the examination immediately<br>D. Administer sedatives or anesthesia independently	**A**
**8**	Which symptom may occur during administration of contrast media?<br>A. Abdominal cramps<br>B. Nausea and vomiting<br>C. Flushing sensation throughout the body<br>D. Only after contrast administration	**C**
**9**	Name the structures at positions 1 and 2?<br>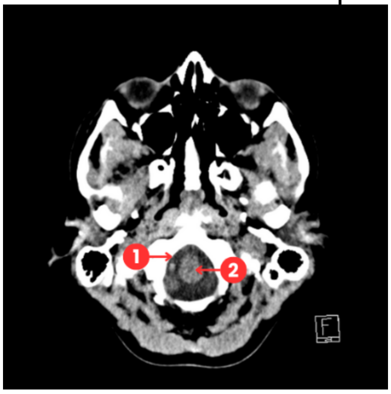<br><br>A. Pons, Medulla oblongata<br>B. Medulla oblongata, Foramen magnum<br>C. Foramen magnum, Pituitary fossa<br>D. Foramen magnum, Medulla oblongata	**D**

Item	Question	Answer
**10**	Name the structure at position 10?<br>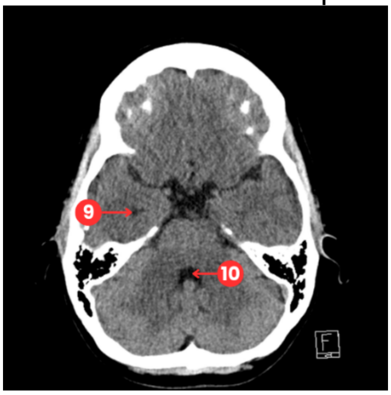<br><br>A. Fourth ventricle<br>B. Third ventricle<br>C. Lateral ventricle<br>D. Midbrain	**A**
**11**	Name the structure at position 3?<br>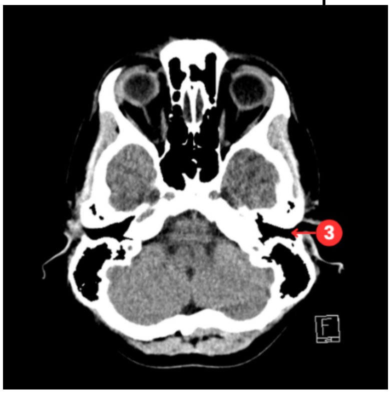<br><br>A. Transverse sinus<br>B. External auditory meatus (EAM)<br>C. Suprasellar cistern<br>D. Quadrigeminal cistern	**B**
**12**	In patients requiring contrast media injection, the serum creatinine level should not exceed how many mg%?<br>A. 1<br>B. 1.5<br>C. 1.73<br>D. 2	**B**
**13**	Which patient position is correct for CT brain with contrast?<br>A. Prone position, feet first<br>B. Supine position, feet first<br>C. Prone position, head first<br>D. Supine position, head first	**D**

Item	Question	Answer
**14**	What is the flow rate for contrast injection in CT brain venous phase?<br>A. 1 mL/sec<br>B. 1.2 mL/sec<br>C. 1.5 mL/sec<br>D. 2 mL/sec	**A**
**15**	What volume of contrast media is required to achieve brain enhancement?<br>A. 20 mL<br>B. 30 mL<br>C. 50 mL<br>D. 60 mL	**C**
**16**	The center of the table height should be positioned at?<br>A. Internal auditory meatus (IAM)<br>B. External auditory meatus (EAM)<br>C. Vertex of the skull<br>D. Supraorbital ridge	**B**
**17**	Extravasation is defined as which condition?<br>A. Contrast leakage outside the vein<br>B. Contrast leakage outside the artery<br>C. Contrast leakage outside cerebral vessels<br>D. Contrast leakage outside coronary vessels	**A**
**18**	Which step helps prevent extravasation?<br>A. Administer preventive medication before contrast injection<br>B. Administer pre-medication for allergy before contrast injection<br>C. Test needle tip position with normal saline before contrast injection<br>D. Test needle tip position with a small amount of contrast before injection	**C**
**19**	Why should patients be instructed not to swallow saliva during contrast injection?<br>A. Movement may cause blurred images<br>B. May cause bitter taste and nausea<br>C. Both a and b are correct<br>D. None are correct	**B**
**20**	Pre-medication must be administered at least how many minutes before contrast injection?<br>A. 15 minutes<br>B. 20 minutes<br>C. 25 minutes<br>D. 30 minutes	**D**
